# Discovery of osmotic sensitive transcription factors in fish intestine via a transcriptomic approach

**DOI:** 10.1186/1471-2164-15-1134

**Published:** 2014-12-18

**Authors:** Marty Kwok-Shing Wong, Haruka Ozaki, Yutaka Suzuki, Wataru Iwasaki, Yoshio Takei

**Affiliations:** Atmosphere and Ocean Research Institute, The University of Tokyo, Tokyo, Japan; Department of Computational Biology, Graduate School of Frontier Sciences, The University of Tokyo, Tokyo, Japan; Department of Biological Sciences, Graduate School of Science, The University of Tokyo, Tokyo, Japan

**Keywords:** Transcriptome, Fish osmoregulation, Intestine, Seawater acclimation, Transcription factors, CEBPB, CEBPD, SGK1, TSC22D3

## Abstract

**Background:**

Teleost intestine is crucial for seawater acclimation by sensing osmolality of imbibed seawater and regulating drinking and water/ion absorption. Regulatory genes for transforming intestinal function have not been identified. A transcriptomic approach was used to search for such genes in the intestine of euryhaline medaka.

**Results:**

Quantitative RNA-seq by Illumina Hi-Seq Sequencing method was performed to analyze intestinal gene expression 0 h, 1 h, 3 h, 1 d, and 7 d after seawater transfer. Gene ontology (GO) enrichment results showed that cell adhesion, signal transduction, and protein phosphorylation gene categories were augmented soon after transfer, indicating a rapid reorganization of cellular components and functions. Among >50 transiently up-regulated transcription factors selected via co-expression correlation and GO selection, five transcription factors, including CEBPB and CEBPD, were confirmed by quantitative PCR to be specific to hyperosmotic stress, while others were also up-regulated after freshwater control transfer, including some well-known osmotic-stress transcription factors such as SGK1 and TSC22D3/Ostf1. Protein interaction networks suggest a high degree of overlapping among the signaling of transcription factors that respond to osmotic and general stresses, which sheds light on the interpretation of their roles during hyperosmotic stress and emergency.

**Conclusions:**

Since cortisol is an important hormone for seawater acclimation as well as for general stress in teleosts, emergency and osmotic challenges could have been evolved in parallel and resulted in the overlapped signaling networks. Our results revealed important interactions among transcription factors and offer a multifactorial perspective of genes involved in seawater acclimation.

**Electronic supplementary material:**

The online version of this article (doi:10.1186/1471-2164-15-1134) contains supplementary material, which is available to authorized users.

## Background

Osmoregulation is an important topic in fish physiology. Bony fishes maintain their body fluid osmolality approximately one-third that of seawater (SW) and therefore they constantly lose water and gain ions in SW but gain water and lose ions in fresh water (FW). Osmoregulation consumes a high proportion of daily energy expenditure in teleosts as they either actively excrete excess ions in SW or take up ions in FW against the respective concentration gradients [1]. The gills, kidney, and intestine are major osmoregulatory organs and play different roles to maintain body fluid homeostasis in both FW and SW [2, 3]. SW teleosts drink copiously and the gastrointestinal tract is responsible for water absorption to compensate for the water loss by osmosis [4, 5]. Although the intestine is an internal organ, its lumen directly contacts environmental water upon drinking in teleost fishes. Osmosensing in fish is accomplished by a combination of sensors in the central nervous system and peripheral osmoregulatory epithelia such as gill, nasal cavity, and intestine [6]. A reflex inhibition in drinking was demonstrated in eel intestine in response to Cl^-^ ions (but not Na^+^) in ingested fluid, indicating the presence of a Cl^-^ specific sensor in eel intestine [7].

Euryhaline fishes that are able to acclimate in both FW and SW transform their intestines dramatically to fulfill the appropriate osmoregulatory roles. When the eel is transferred from FW to SW, the intestinal wall decreases in thickness, and the anterior intestine became highly vascularized through angiogenesis within 1–2 days [8]. Monovalent ions (Na^+^, Cl^-^) in the imbibed SW are actively absorbed while divalent ions (Ca^2+^, Mg^2+^, SO4^2-^) are precipitated to decrease luminal fluid osmolality [5, 9]. The composition of epithelial transporters is also reorganized extensively. An upregulation of mucosal Na-K-2Cl cotranspoter (NKCC2/ SLC12A1) remarkably increases the ion absorption rate, which is facilitated by the serosal Na-K-ATPase and Na^+^-bicarbonate exchanger [10]. The rapid and efficient ion absorption mechanism in teleost intestine is unique in vertebrates [11]. The SW teleost intestine is an absorptive epithelium that is similar to the thick ascending loop of Henle in mammalian nephron, and thus was often used as a comparative model for the study of kidney tubules, especially in the study of transporter mechanisms and cellular regulation in response to volume and salt stresses [12]. Several hormones such as cortisol, prolactin, growth hormone, atrial natriuretic peptide, arginine vasotocin, guanylin, and vasoactive intestinal peptide regulate transepithelial ion transport [13–20]. However, the transcription factors that govern hormone actions, cell proliferation, apoptosis, angiogenesis, transporter metabolism etc., are unclear.

Transcriptomic approaches have been used to investigate the dynamics osmoregulatory organ function of teleosts [21–24]. However, microarray- or pyrosequencing-based transcriptomic reads do not provide sufficient depth and coverage for the detection and quantitation of low expression genes, which may result in a biased discovery towards high expression genes. In the present study, the objective was to discover the transcription factors that are responsive to the SW challenge in medaka intestine. Illumina HiSeq Sequencing was selected to provide a deep coverage of identified genes as the large number of reads allows quantification of gene expression by mapping to reference genome, and is usually sufficient to detect most expressed gene even at low expression level [25], which is an ideal choice at a cost-performance perspective. Medaka was used owing to the relative completeness of the genome data, which can streamline the RNA-seq analysis and guarantee high accuracy and reliability. Medaka is also a euryhaline species that can survive a direct FW to 50% SW (ca. twice hypertonic to plasma) transfer [26]. We combined physiological and bioinformatic approaches in the experimental design, in which FW medaka was challenged by 50% SW transfer and time-dependent changes in intestinal transcriptome were analyzed by RNA-seq. We focused on the early upregulated transcription factors that could initiate subsequent intestinal transformations and lead to altered function from FW to SW.

## Results

### Illumina sequencing and reference gene mapping

Illumina 101 bp paired end sequencing were performed on all 25 intestine samples collected from medaka after 0 h, 1 h, 3 h, 1d, and 7 d after SW transfer (N = 5) The sequenced reads ranged from 17.3 to 60.5 million reads with average 37.9 million reads in each sample (Table 1). The read were mapped against the annotated genome of Medaka. More than 83% of the sequences were successfully mapped sequences, indicating the data sets are representative.

**Table 1:** **Illumina sequencing and mapping statistics**

Sample	Treatment	Number of reads	Number of mapped	Number of unique read	Number of multiple	Number of unmapped	% mapped	% unique	% concordantly mapped pair/mapped pair
medaka_DT1	FW	45,187,196	38,886,015	36,800,226	2,085,798	6,301,181	86.06	81.44	96.04
medaka_DT2	FW	60,510,650	51,531,207	48,089,685	3,441,522	8,979,443	85.16	79.47	96.32
medaka_DT3	FW	49,132,444	42,149,176	39,787,090	2,362,086	6,983,268	85.79	80.98	96.01
medaka_DT4	FW	53,098,918	45,320,354	42,439,720	2,880,634	7,778,564	85.35	79.93	96.12
medaka_DT5	FW	42,600,708	36,530,248	34,556,499	1,973,749	6,070,460	85.75	81.12	96.46
medaka_DT6	SW1h	42,628,084	36,663,691	34,708,187	1,955,504	5,964,393	86.01	81.42	95.96
medaka_DT7	SW1h	56,932,366	48,827,425	46,194,052	2,633,373	8,104,941	85.76	81.14	96.90
medaka_DT8	SW1h	42,093,406	36,151,083	34,098,610	2,052,473	5,942,323	85.88	81.01	96.47
medaka_DT9	SW1h	52,158,762	44,893,371	42,420,952	2,472,419	7,265,391	86.07	81.33	96.05
medaka_DT10	SW1h	36,707,006	31,548,390	29,929,884	1,618,506	5,158,616	85.95	81.54	96.06
medaka_DT11	SW3h	41,952,606	36,052,499	34,168,827	1,883,672	5,900,107	85.94	81.45	95.05
medaka_DT12	SW3h	38,901,344	33,331,036	31,561,823	1,769,213	5,570,308	85.68	81.13	96.60
medaka_DT13	SW3h	48,830,644	42,173,994	39,875,418	2,298,576	6,656,650	86.37	81.66	94.54
medaka_DT14	SW3h	43,038,240	37,145,021	35,132,181	2,053,293	6,445,624	85.80	81.28	96.36
medaka_DT15	SW3h	45,401,548	38,955,924	36,902,631	2,053,293	6,445,624	85.80	81.28	96.36
medaka_DT16	SW1d	41,811,642	35,924,612	34,060,638	1,863,974	5,887,030	85.92	81.46	96.24
medaka_DT17	SW1d	27,329,496	23,684,730	22,440,772	1,243,958	3,644,766	86.66	82.11	95.70
medaka_DT18	SW1d	26,278,316	22,719,337	21,476,111	1,243,226	3,558,979	86.46	81.73	95.82
medaka_DT19	SW1d	20,440,212	20,139,877	19,080,869	1,059,008	3,300,335	85.64	81.09	96.01
medaka_DT20	SW7d	23,440212	20,139,877	19,080,869	1,059,008	3,300,335	85.92	81.40	96.56
medaka_DT21	SW7d	20,399,688	17,475,541	16,546,834	928,707	2,924,147	85.67	81.11	96.54
medaka_DT22	SW7h	17,289,434	14,563,145	13,793,496	769,649	2,726,289	84.23	79.78	96.92
medaka_DT23	SW7h	23,527, 904	19,851,220	18,617,357	1,233,863	3,676,684	84.37	79.13	97.13
medaka_DT24	SW7h	27,563,352	23,083,585	21,713,537	1,370,048	4,479,767	83.75	78.78	97.48
medaka_DT25	SW7h	21,133,038	18,209,017	17,192,056	1,016,961	2,924,021	86.16	81.35	96.18

DT: digestive tract; FW: freshwater; SW: 50% seawater.

### Gene ontology analysis

GO terms enriched in the 1 h-after-SW transfer group are listed in Table 2. According to biological process, protein phosphorylation, regulation of DNA-dependent transcription, cell adhesion, and signal transduction were highly ranked in GO enrichment. According to molecular function, protein binding, protein tyrosine kinase activity, protein serine/threonine kinase activity, and actin binding were enriched. According to cellular component, significant enrichment in GO terms was found in integrin complex and cytoskeleton.

**Table 2:** **Gene ontology enrichment analysis on the transcriptomes of FW vs SW 1 h in medaka intestine (p < 0.05)**

	Biological process		Molecular function		Cellular component	
Rank	Annotation	p-value	Annotation	p-value	Annotation	p-value
1	protein phosphorylation	1.90E-10	protein binding	8.60E-13	integrin complex	1.60E-03
2	regulation of transcription, DNA-dependent	1.10E-08	protein tyrosine kinase activity	8.10E-08	extra cellular region	7.50E-03
3	cell adhesion	4.20E-05	protein serine/threonine kinase activity	1.70E-05	Cytoskeleton	8.60E-03
4	regulation of small GTPase mediated signal transduction	2.20E-04	actin binding	4.20E-05	proteinaceous extracellur matri	2.30E-02
5	signal transduction	2.20E-04	sequence-specific DNA binding transcripttion factor activity	5.30E-05		
6	integrin-mediated signaling pathway	1.01E-03	guanyl-nucleotide exchange factor activity	5.70E-04		
7	intracellular signal transduction	1.72E-03	phospholipid binding	1.35E-03		
8	glucose metabolic process	1.94E-03	sequence-specific DNA binding	2.12E-03		
9	cytokine-mediated signaling pathway	2.27E-03	non-membrane spanning protein tyrosine kinase activity	2.69E-03		
10	peptidy I-tyrosine dephosphorylation	2.43E-03	cytokine receptor activity	2.69E-03		
11	cell cycle arrest	4.30E-03	ATP binding	3.86E-03		
12	multicellular organismal development	5.18E-03	G-protein coupled peptide receptor activity	3.90E-03		
13	G-protein coupled receptor signaling pathway	5.90E-03	protein domain specific binding	4.53E-03		
14	neurotransmitter transport	9.35E-03	MAP kinase activity	5.07E-03		
15	intracellular protein kinase cascade	1.75E-02	protien tyrosine/serine/threonine phosphatase activity	5.98E-03		
16	MAPK cascade	1.79E-02	receptor activity	6.97E-03		
17	transmembrane receptor protein tyrosine kinase signaling pat…	1.93E-02	calcium ion binding	7.11E-03		
18	positive regulation of GTPase activity	2.71E-02	protein tyrosine phosphatase activity	1.02E-02		
19	regulation of cellular component biogenesis	2.71E-02	transmembrane receptor protein tyrosine kinase activity	1.62E-02		
20	hormophilic cell adhesion	3.446E-02	GTPase activator activity	1.76E-02		
21	regulation of ARF protein signal transduction	3.48E-10	neurotransmitter: sodium symporter activity	2.08E-02		
22	regulation of cellular component organization	3.48E-02	small GTPase regulator activity	2.20E-02		
23	cell-matrix adhesion	4.77E-02	calmodulin binding	2.65E-03		
24	polyol metabolic process	4.88E-02	organic acid transmembrane transporter activity	2.89E-02		
25			ubiquitin protein ligase activity	2.99E-02		
26			protein complex binding	3.27E-02		
27			phosphotransferase activity, alcohol group as acceptor	3.67E-02		
28			ARF guanyl-nucleotide exchange factor activity	3.92E-02		
29			signal transducer activity	4.66E-02		
30			kinase activity	4.79E-02		

### Transiently upregulated genes

The intestinal transcription-related genes that are involved in the SW acclimation were screened from the genes with early transient increase in expression, which is defined as genes with significant increases in gene expression (one-way ANOVA, Tukey; *p* < 0.05) in 1 h and/or 3 h post-transfer groups compared to those of 0 h, 1d, and 7d. The candidates genes were further filtered to remove low expression genes (<20 RPM at 1 h or 3 h post-transfer). Genes with significant correlation (Pearson r > 0.8) in expression with a known osmotic transcription factor (TSC22D3) among the early transient increase genes were selected for further analysis (Additional file 1: Table S1). The selected genes were further filtered by GO annotation to obtain transcription-related genes and 57 genes were further analyzed by real-time PCR.

### Real-time PCR

**Figure 1:**
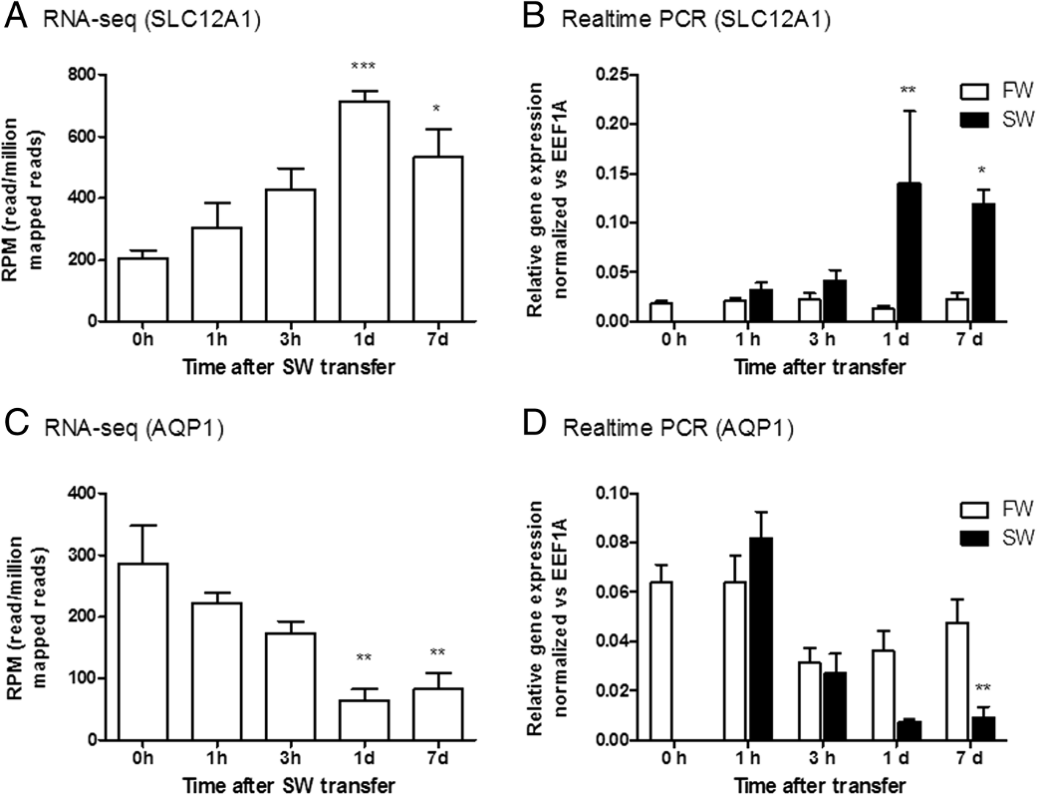
**Representative validation of quantitative transcriptome by realtime PCR.** RNA-seq and realtime PCR results of time course expression profiles of SLC12A1 (**A** and **B**) and AQP1 (**C** and **D**) are shown respectively. Statistical significant groups are indicated by asterisks (one-way ANOVA, Tukey for **A** and **C**; two-way ANOVA, Bonferroni for **B** and **D** with time-matched comparison. *p < 0.05; **p < 0.01; ***p < 0.001).

To confirm the adequacy of the SW transfer stimulus on the medaka intestine, SLC12A1/NKCC2 and AQP1 were used as reference genes since these transporters are well-acknowledged to change in teleost intestine after SW challenges [27–29]. The real-time PCR results of SLC12A1 and AQP1 showed a gradual increase and decrease in gene expression at 1d and 7d post-transfer respectively, which strongly mirrored the RNA-seq results (Figure 1). FW control transfer did not induce significant changes in gene expressions.

**Figure 2:**
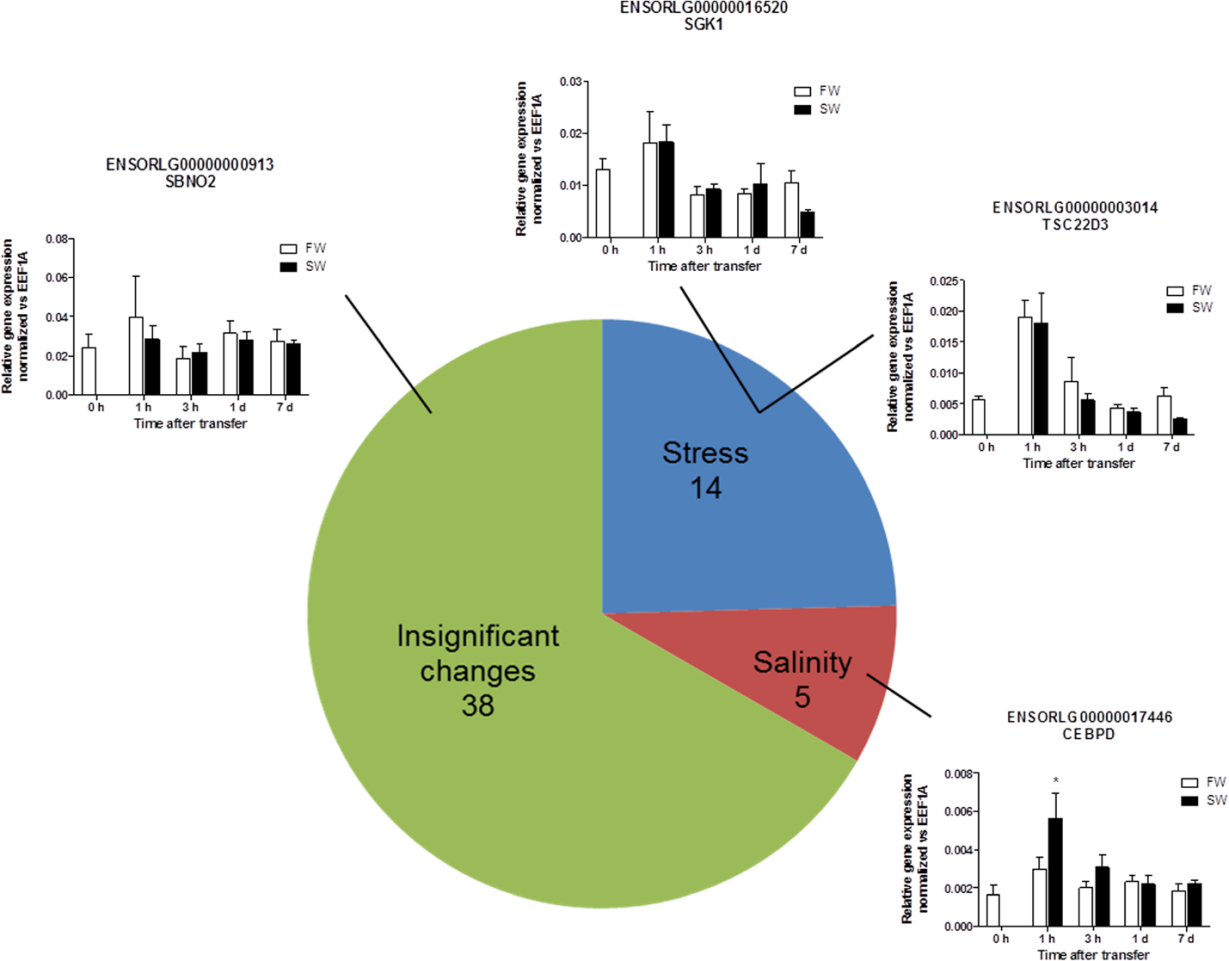
**Summary of validation of hyperosmotic effects on intestinal transcription factors discovered from transcriptome by real-time PCR.** The transcription factors were examined, and 19/57 were reproducible. Among the candidate genes, 5/19 were found to be specific towards hyperosmotic challenge (red) while others were related to general transfer stress (blue). Small graphs are representative real time PCR results of each category: stress-related, TSC22D3 and SGK1; salinity-related, CEBPD; insignificant change, SBNO2. Statistical significant groups are indicated by asterisks in the bar graphs (Two-way ANOVA, Bonferroni with time-matched comparison.*p < 0.05).

The same set of cDNA was subsequently used for validation of transcription factors and 19/57 of the transiently upregulated genes were reproducible (Figure 2). Among the genes in which transient upregulation was confirmed by quantitative PCR, 14/19 were found to be stress-related since similar increases were observed after FW-FW transfer, including TSC22D3 and SGK1 (Figure 2; Additional file 2: Figure S1-S4). However, 5 novel transcription factors, CEBPB, CEBPD, RAD54L2, HIF3A, and LDB1, were discovered to respond specifically to SW challenges, and thus used for further analyses.

### STRING analysis and protein interaction network assembly

**Figure 3:**
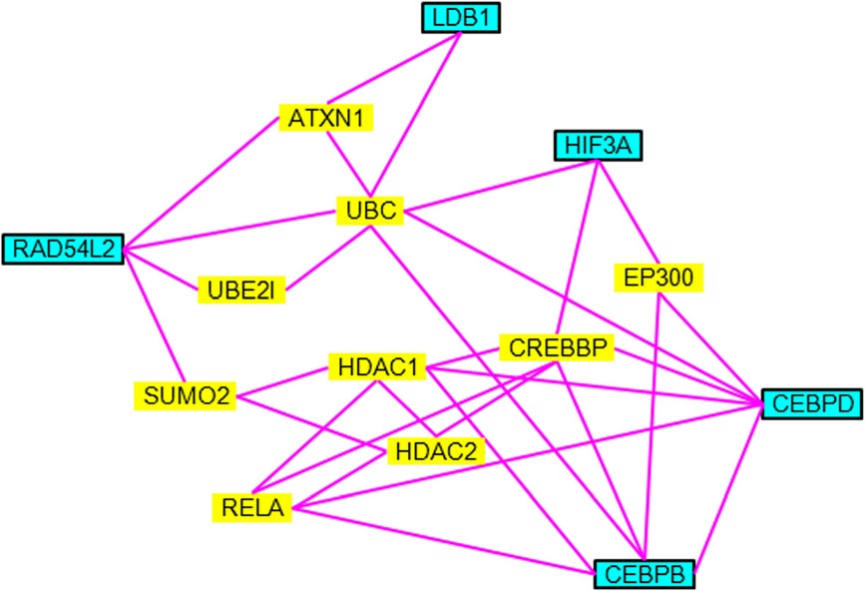
**Protein interaction network of salinity-related transcription factors.** Genes highlighted in blue represent the salinity-related transcription factors discovered from transcriptome studies. Yellow highlighted genes indicate the protein interaction partners extracted from STRING analysis.

**Figure 4:**
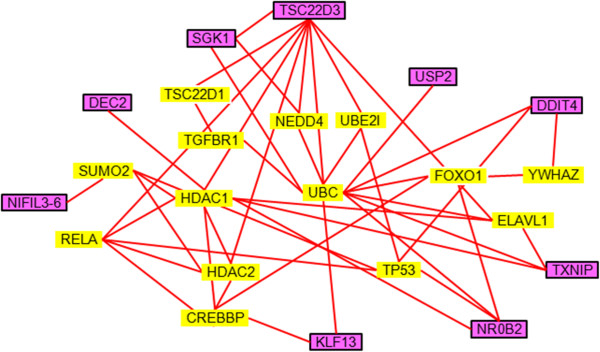
**Protein interaction network of stress-related transcription factors.** Genes highlighted in purple represent the stress-related transcription factors discovered from transcriptome studies. Yellow highlighted genes indicate the protein interaction partners extracted from STRING analysis.

Using the STRING analysis, protein interaction networks of hyperosmotic-related (Figure 3) and stress-related (Figure 4) transcription factors were constructed by overlapping the common interacting protein partners. Stress-related transcription factors such as SGK1, TSC22D3, NR0B2, and DDIT4 interact with the UBC-, TP53-, and RELA-related networks (Figure 4). Hyperosmotic-specific transcription factors such as CEBPB and CEBPD affect a similar protein network including UBC and RELA (Figure 3). EP300, CREBBP, and ATXN1 were only found in salinity-specific network, and these could have specific roles on transforming the intestine from FW- to SW-types.

## Discussion

### GO enrichment

As the focus of the present study is on the early transcription factors that may signal the transformation of intestinal functions, GO comparison was performed between pre-transfer (FW) and 1 h SW transfer groups. The results indicated that the intestine transcriptome responded rapidly to the change in environment salinity and the GO terms enriched were highly relevant to the contemporary understanding of the transformation events occurred in fish intestine when entering SW [5]. GO annotations including signal transduction, regulation of transcription, protein phosphorylation, regulation of gene expression were all markedly enriched, indicating neuron- or hormone-mediated signals were commanding the structural and functional changes. For cellular function, actin binding, and cell adhesion enhancement may indicate that the cells adaptively respond to the cellular dehydration by adjusting the membrane permeability and cytoplasmic osmolytes to increase survival. Markers of cell cycle arrest were enriched and suggested an increase in apoptosis and recruitment of new cells to proliferate in SW. Glucose metabolic process and ATP-binding enrichment indicated a high energy turnover following adrenergic response to cope with the abrupt transfer stress, and to provide energy for cell homeostasis. Augmentation in peptide receptor and kinase activities indicated a role of endocrine stimulation in the transformation including the various changes in ion transporter expressions. The enrichment in integrin complex and cytoskeleton also indicates stress responses at both membrane and cytoplasm levels, which could be related to cellular dehydration consequent to imbibition of SW. Altogether, these changes are expected during transformation of intestinal functions from FW- to SW-types, which include apoptosis of old cells and recruitment of new cells, reorganization of transporters for ion/water absorption, and changes in para-cellular permeability.

### Validation of seawater treatment effects

In order to validate the accuracy and reproducibility of current transcriptome analyses, genes that are known to change consistently after SW transfer were compared between transcriptome and real-time PCR data. The NKCC2 expression increased while AQP1 expression decreased similarly in both analyses (Figure 1), indicating high reliability of the current analyses. The results confirmed the reproducibility of the SW transfer effects and thus allow the extrapolation of the data sets for the discovery of novel genes related to SW acclimation. The increased NKCC2 gene expression was consistent with the increased ion absorption in SW teleost intestine [27, 28]. However, the decreased AQP1 expression in medaka intestine after SW transfer was puzzling since SW acclimation up-regulated AQP1 expression in the eel intestine but down-regulated that in seabream [30–32]. The down-regulation of AQP1 in medaka intestine could be similar to that of seabream, but the physiological implication of this species-specific difference is not clear because a high demand for water absorption by the intestine of SW teleost is expected. A search for expression pattern of other AQP members in the transcriptome data was performed but all known AQPs in medaka did not increase their expression levels in SW (data not shown), thus ruling out the possibility that some other AQP members may have replaced the function of AQP1 in medaka intestine. The species-specific difference in AQP1 expression may also indicate that the various intestinal ion transporting mechanisms could be present in different teleost lineages.

### Novel transcription factors for osmoregulation

CEBPB and CEBPD are important transcriptional activators in the immune and inflammatory responses via cytokines such as interleukin 6 [33]. High salt intake increased the transcription of angiotensinogen by decreasing the methylation around the CEBP binding sites at the promoter region [33], thus CEBP may contribute to the control of the renin-angiotensin system at the transcriptional level. Multiple CEBP binding sites are present on the promoter region of NKCC2 in mice [34]. CEBP binding sites in the promoter region control the gene expression of cystic fibrosis transmembrane conductance regulator (CFTR) in human under basal and cAMP-stimulated transcription states [35]. In addition, the protein interaction network identified in this study indicates possible involvement of a cAMP-related pathway, represented by CREBBP, in the initial signaling in SW intestine transformation. Therefore, CEBP binding sites at the promoter regions of ion transporters will be of high interest in the future studies.

RAD54L2 is a DNA helicase that modulates androgen receptor-dependent transactivation in a promoter-dependent manner. The osmoregulatory function of this helicase is unknown and it is possibly one of the glucocorticoid receptor regulatory elements in fish, which facilitate or mediate the mineralocorticoid actions of cortisol. HIF3A is known to be involved in the adaptive response to hypoxia and interacts with a number of stress-related proteins including HSP90. The SW challenge may have increased energy consumption for reorganization of cellular components, which led to cellular hypoxia. LDB1 was recently regarded as a master gene for the control of red blood cell development [36]. The possible role of LDB1 could be as a regulator of KLF1, which in turn modulates the expressions of water channel AQP1 and cell adhesion molecule CD44 [37]. Therefore, LDB1 could regulate expression of transporters and act as an upstream controller for cell adhesion and cytoskeleton as indicated in the GO analysis.

Besides the 5 salinity-specific transcription factors discovered in this study, ATXN1, EP300, and CREBBP, which are independent of the stress-related interacting proteins, were also singled out from the protein interaction networks (Figures 3 and 4). The functional role of ATXN1 in mammals is not clear but our results suggest that ATXN1 directly interacts with salinity-specific transcription factors LDB1 and RAD54L2, which could be a novel regulatory factor under salt stress. CREBBP is an important mediator in the cAMP signaling. In fact, the cAMP pathway was shown to be involved in the osmoregulatory sensing and functions in teleosts [38–41]. EP300 is a tumor-related transcription factor that is involved in restraining cell growth and division. EP300 also mediates cAMP-gene regulation by specific binding to phosphorylated CREBBP and acts as a co-activator of HIF1A in mammals [42]. It is possible that the EP300 in fish may interact with HIF3A as suggested by out protein interaction network.

### Stress-related transcription factors

Previous studies have demonstrated that TSC22D3 and serum/glucocorticoid regulated kinase (SGK1) are osmotic stress regulators in the gill of several fish models including killifish, medaka, eel, and tilapia [43–49]. In medaka intestine, however, we found that their upregulation soon after SW transfer is partly due to handling stress as shown by the real time PCR results in FW control transfer (Figure 2). The discrepancy could be due to the difference in tissues (gill vs. intestine) but we noticed that previous studies were lacking proper time-dependent control transfer, and where the salinity effects were concluded by the comparison between pre-transfer and post-transfer groups [24]. Another possibility is that some of these stress-related transcription factors are also involved in the regulation of ion transport, amelioration of cellular stress such as DNA damage, and mobilization of energy via adrenergic stimulation. In fish opercular epithelium, α- and β-adrenergic stimulation can inhibit and stimulate ion transport via Ca^2+^/IP_3_ and cAMP signaling pathways respectively [50].

The overlapping of stress- and salinity-related transcription factor interaction networks infers an intimate relationship between general emergency responses and osmoregulation (Figures 3 and 4). In fact, the effects of handling stress on the perturbation of hydromineral balance and osmoregulatory functions in fish were documented [43, 51]. During an emergency, as induced by handling, fish inevitably increase their ventilation rate to obtain sufficient oxygen to meet the energy demand. However, the increase in ventilation also elevates ion and water fluxes, disrupting the equilibrium of ion absorption/excretion [51]. Activity or expression of Na-K-ATPase and Na-K-2Cl co-transporter changed in accordance to the changes of plasma osmolality induced by handling stress [43]. Stress undoubtedly increases metabolic demand, and it was argued that the metabolic performance of fish is limited by osmoregulatory cost [52]. In the light of these findings, some of the stress-related transcription factors could also be related to the perturbation of hydromineral balance.

Previous studies demonstrated a role of SGK1 in salinity acclimation via controlling the activities of ion transporters in fish [45, 53]. SGK1 expression is ubiquitous and upregulated by a large number of stimuli including hyperosmotic or isotonic cell shrinkage, excessive glucose concentration, mechanical stress, metabolic acidosis, oxidative stress, heat shock, and DNA damage [54]. Leptin was shown to promote glucose mobilization during SW transfer in tilapia [55], indicating a hormone-driven hyperglycemia under a hyperosmotic challenge. Therefore, it is possible that emergency responses, such as an increase in plasma glucose, may lead to an artefactual increase in SGK1 expression. SGK1 is a powerful modulator for gene expression and phosphorylation of a large number of ion transporters such as Na-K-ATPase, NKCC2, and CFTR [56]. In addition, SGK1 is highly expressed in tumor cells and it phosphorylates EP300 to acetylate NF-kB [57] and is downregulated by ubiquitination via NEDD4-2 [58]. Our protein interaction network (Figure 4) also suggested a wide spread interactions of SGK1 with other transcription factors, in particular with TSC22D3. TSC22D3 was firstly suggested to be acting as a osmotic-stress transcription factor 1 (Ostf1) [1] as the expression was upregulated by hyperosmotic challenge and the protein is localized in ionocytes of the teleost gills [44, 59]. However, recent studies suggested a wide spectrum of TSC22D3 functions in developmental regulation, reproduction, inflammation, and tumor suppression [60, 61], and it may play a major role as a glucocorticoid-induced leucine zipper. Both SGK1 and TSC22D3 are mediators of glucocorticoids and thus their roles in osmoregulation could be related to the release of cortisol, which acts as the mineralocorticoid as well as the glucocorticoid in teleosts [62]. Therefore, their functions may not be directly specific to osmotic stimulus but rather related to the pathway dependent stimulation.

Other stress-related genes such as DDIT4 and TXNIP are negative regulators to cellular stress and were stimulated by glucose increment [63]. NR0B2, also known as small heterodimer partner, is an orphan nuclear receptor that represses transcription and interacts with EP300 [64]. KLF13 is also a transcription repressor that binds to basic-transcription element [65]. From the individual gene function to the interaction network, these stress-related transcription factors play vital roles in cell survival and emergency preparation, which is important under both general and osmotic stresses.

## Conclusion

**Figure 5:**
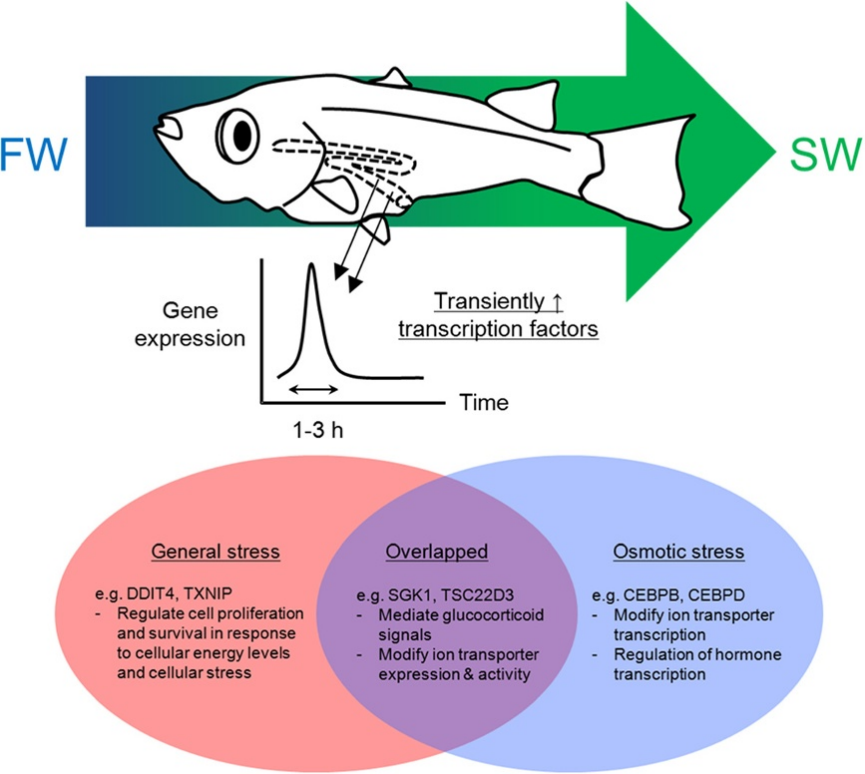
**Graphical summary of the key findings.** The early transiently-upregulated transcription factors can be categorized into two groups: general stress vs osmotic stress. Among these transcription factors, some are common to the two groups and could be important in glucocorticoid-mediating functions.

Our work for the first time investigated quantitatively the transcriptome of fish intestine in response to SW transfer in a time-course manner. This bottom-up approach has allowed the discovery of novel transcription factors involved in the osmoregulatory response to SW challenge. Results of GO analysis showed that the transcriptome analysis revealed representative changes and the data sets are valuable for discovering novel genes involved in salinity acclimation in fish. The protein interaction networks of the salinity- and stress-related genes indicate overlapping of the transcription factors between emergency and SW acclimation (see Figure 5 for summary). The newly-discovered salinity-related transcription factors provide important directions on the studies in osmoregulation and its regulation in fishes.

## Methods

### Animal husbandry

Medaka (*Oryzias lapties*), HdrR strain, were bred and kept in a freshwater (FW) recirculating aquarium system in the Atmosphere and Ocean Research Institute, The University of Tokyo. The aquarium system was controlled at 26°C with a photoperiod of 14 h/10 h light/dark cycle. Fish were fed daily with freshly hatched brine shrimp. Individuals (1.0 - 1.2 g body weight) were randomly divided into nine experimental tanks (N = 6 in each group). Four groups were transferred to FW (control-transfer) and another four groups were transferred to 50% seawater (SW) for 1 h, 3 h, 1 d, and 7 d, respectively. One group remained undisturbed to serve as a pre-transfer control. In sampling, the experimental fish was anaesthetized in 0.1% tricaine methanesulfonate (MS-222) and then sacrificed by spinal transection. The intestine was immediately dissected, snap-frozen in liquid nitrogen, and stored at -80°C until use. Experiments were repeated twice to obtain sufficient RNA samples for transcriptome and real-time PCR analyses. All animal experiments were carried out according to the ethical guidelines and protocols approved by the Animal Experiment Committee of the University of Tokyo.

### RNA sequencing

Total RNA was extracted from the intestine samples using Isogen (Nippon Gene, Toyama) according to the manufacturer’s protocols. RNA quality was monitored using an Agilent 2100 Bioanalyzer system (Agilent Technology, CA ) and only RNA samples with RNA integrity number values over 7.0 were used for sequencing. The cDNA libraries were prepared from the RNA samples of 5 treatment groups (FW, SW 1 h, 3 h, 1 d, and 7 d; N = 5 in each case) using TruSeq RNA Sample Preparation v2 (Illumina Inc., CA), and were sequenced by Illumina HiSeq 2500 (101 bp, paired-end) in the Laboratory of Functional Genomics, The University of Tokyo, according to the manufacturer’s protocols. From the Illumina chastity filter analysis, the percentages of reads that passed the filter (PF reads), the percentage of bases with > = Q30 in the PF reads, and the mean quality score of the PF reads were of 96.06 ± 0.59, 93.78 ± 0.83, and 36.23 ± 0.26, respectively. We performed the FastQC analysis on the PF reads, uniquely mapped reads, and unmapped reads. The percentage of reads with mean sequence quality > = Q30 was 94.32 ± 1.21, 96.76 ± 1.02, and 83.52 ± 1.58, respectively, indicating that the quality of the unmapped reads was lower than that of the uniquely mapped reads (Additional file 3: Table S2).

### Reference genome sequence and annotation data

The medaka HdrR genome sequence was obtained from UCSC Genome Browser [oryLat2, http://genome.ucsc.edu/]. The annotated gene models (NCBIM37) and the annotation of rRNAs were taken from Ensembl [release 69, http://www.ensembl.org/]. The annotations of tRNAs were retrieved from UCSC Genome Browser [http://genome.ucsc.edu/].

### RNA-seq data analysis

The RNA-seq data were mapped to the medaka genome using TopHat (version 2.0.9) with default parameters [66]. Sequence reads that were mapped to multiple genes or positions were removed. HTSeq (version 0.5.3p9) were used to count the number of reads mapped on each gene [67]. For normalization, the count for each gene was divided by the number of million uniquely mapped reads in each sample (RPM; read per million mapped reads). Genes that did not have more than five normalized counts in at least two samples were removed from further analyses.

### Gene ontology (GO) analysis

The GO terms of all transcripts in SW 1 h group were compared with those of FW group using topGO package (version 2.14.0) in Bioconductor [http://www.bioconductor.org/] with the weight01 algorithm to calculate an enrichment score for each gene ontology term [68]. Significantly-enriched GO terms were ranked using the *p*-values with threshold at *p* < 0.05.

### Gene expression profile analysis

To extract the transcription-related genes that are involved in the SW acclimation in the intestine, genes with early transient increase in expression were screened. Early transient increase is defined as significant increase in gene expression (one-way ANOVA, Tukey; *p* < 0.05) in 1 h and/or 3 h post-transfer groups compared to those of 0 h, 1d, and 7d. As the number of genes that fell in this category was too large for further analysis, we further narrowed the candidates by filtering genes with <20 RPM at 1 h or 3 h post-transfer. The expression profile of a representative gene (TSC22D3) in the early transient increase category was used to search for co-expressed genes in the transcriptome using Pearson’s correlation and genes that exhibited *r* >0.8 among the 25 samples were selected for further analysis. Gene description and GO annotation of these genes were obtained at Uniprot Knowledge Database. Genes with “transcription” and/or “DNA-binding” in the description or GO terms were screened and further analyzed by real-time PCR.

### Real-time PCR

To validate the hyperosmotic effects that were discovered in transcriptome analysis, we performed real-time PCR on the selected genes using a separate set of RNA samples from a duplicate experiment as described in the previous section. We included control transfer (FW to FW) samples to check whether the observed effects are salinity-specific or are due to simply the physical transfer (N = 6 at each time point). Total RNA was extracted and subsequently treated with DNase I (Life Technologies, CA) to remove genomic DNA and 1 μg of the treated RNA was reverse-transcribed with Iscript cDNA Synthesis Kit (Bio-Rad, CA) according to the manufacturer’s protocols. Real-time PCR was performed in 10 μL reactions using Kappa SYBR 2X PCR mix (Kappa Biosystems, MA) and ABI 7900HT Fast Real Time PCR System (Life Technologies, CA). The amplification of a single amplicon was confirmed by analyzing the melting curve after the real-time cycling. Elongation factor 1 alpha (EEF1A) was used as an internal control to normalize the gene expressions among different samples. Our transcriptome data also indicated a stable expression of EEF1A among all samples (data not shown), as expected for an internal control housekeeping gene. Ion transporters such as Na-K-Cl co-transporter (SLC12A1) and aquaporin (AQP1) were included as positive controls for SW transfer effects on the intestine. Relative expression of target genes was quantified by the 2–[delta][delta]Ct method where [delta][delta]Ct = [delta]Ct,target - [delta]Ct,EEF1A. Real time PCR primer sequences are listed in Additional file 4: Table S3.

Gene expressions in intestine at various times following FW-FW and FW-SW transfers were analyzed by two-way ANOVA followed by Bonferroni’s multiple-comparison test. Time-matched group comparison was made and groups with *p* < 0.05 were considered as significantly different (GraphPad Prism Ver. 5.0 for Windows, CA). Genes with increased expression in respond to SW transfer but not FW transfer 1 h and/or 3 h while insignificant changes at other time points were considered as salinity-specific. Genes with increase expression in respond to both FW and SW transfer at 1 h and/or 3 h while insignificant changes at other time points were considered as stress-related.

### Protein-protein interaction by STRING analysis

After validation of transiently upregulated genes by real-time PCR, the selected transcription factors were further divided into 3 categories (see real-time PCR results). The transcription factors specific to handling stress or SW challenge were searched for potential interacting proteins using the Known and Predicted Protein-Protein Interaction Database (STRING 9.05). The interacting proteins were extracted from the database using human as the model organism since the database coverage is higher than those of medaka or zebrafish. Protein interaction networks were constructed by connecting the overlapped genes among the query genes and interacting proteins.

### Data availability

The sequencing data sets for the RNA-seq are available from the DDBJ/EBI/NCBI databases with accession number DRP002295.

## Electronic supplementary material

Additional file 1: Table S1: Early transient upregulated genes after a time-course SW transfer (1 h and/or 3 h vs 0 h, 1d, and 7d; one-way ANOVA, Tukey; p < 0.05). (TIFF 646 KB)

Additional file 2: Figures S1-S4: Real time PCR results of the transcription factors in medaka intestine discovered from transcriptome. The expression patterns were categorized to salinity-related, stress-related, and insignificant changes. Statistical significant groups are indicated by asterisks in the bar graphs (two-way ANOVA, Bonferroni with time-matched comparison.*p < 0.05). (ZIP 666 KB)

Additional file 3: Table S2: Quality controls using Casava and FastQC. % ≥ Q30: the percentages of reads whose mean sequence quality were ≥ Q30. DT: digestive tract; FW: freshwater; SW: 50% seawater. (TIFF 209 KB)

Additional file 4: Table S3: Primer sequences for real time PCR. (TIFF 546 KB)

## Abbreviations

AQP1: Aquaporin 1
ATXN1: Ataxin 1
cAMP: cyclic-AMP
CD44: CD44 Molecule
CEBP: CCAAT/Enhancer Binding Protein (C/EBP)
CEBPB: CCAAT/Enhancer Binding Protein (C/EBP), Beta
CEBPD: CCAAT/Enhancer Binding Protein (C/EBP), Delta
CFTR: Cystic Fibrosis Transmembrane Conductance Regulator
CREBBP: CREB Binding Protein
DDIT4: DNA-Damage-Inducible Transcript 4
EEF1A: Eukaryotic Translation Elongation Factor 1 Alpha 1
EP300: E1A Binding Protein P300
GO: Gene Ontology
HIF1A: Hypoxia Inducible Factor 1, Alpha Subunit
HIF3A: Hypoxia Inducible Factor 3, Alpha Subunit
HSP90: Heat Shock Protein 90
IP_3_: Inositol trisphosphate
KLF1: Kruppel-Like Factor 1 (Erythroid)
KLF13: Kruppel-Like Factor 13
LDB1: LIM Domain Binding 1
MS-222: Tricaine methanesulfonate
NKCC2/SLC12A1: Na-K-2Cl Cotransporter
Solute Carrier Family 12: Member 1
NR0B2: Nuclear Receptor Subfamily 0, Group B, Member 2
RAD54L2: Androgen Receptor-Interacting Protein 4
RELA: V-Rel Avian Reticuloendotheliosis Viral Oncogene Homolog A1
RNA-seq: RNA sequencing
SGK1: Serum/Glucocorticoid Regulated Kinase 1
TP53: Tumor Protein P53
TSC22D3: TSC22 Domain Family, Member 3
TXNIP: Thioredoxin Interacting Protein
UBC: Ubiquitin C.
