# Supplementary figures and images for: Discovery of osmotic sensitive transcription factors in fish intestine via a transcriptomic approach
Source: BMC Genomics. 2014 Dec 18;15(1):1134. doi: 10.1186/1471-2164-15-1134 (PMC4377849; doi:10.1186/1471-2164-15-1134)

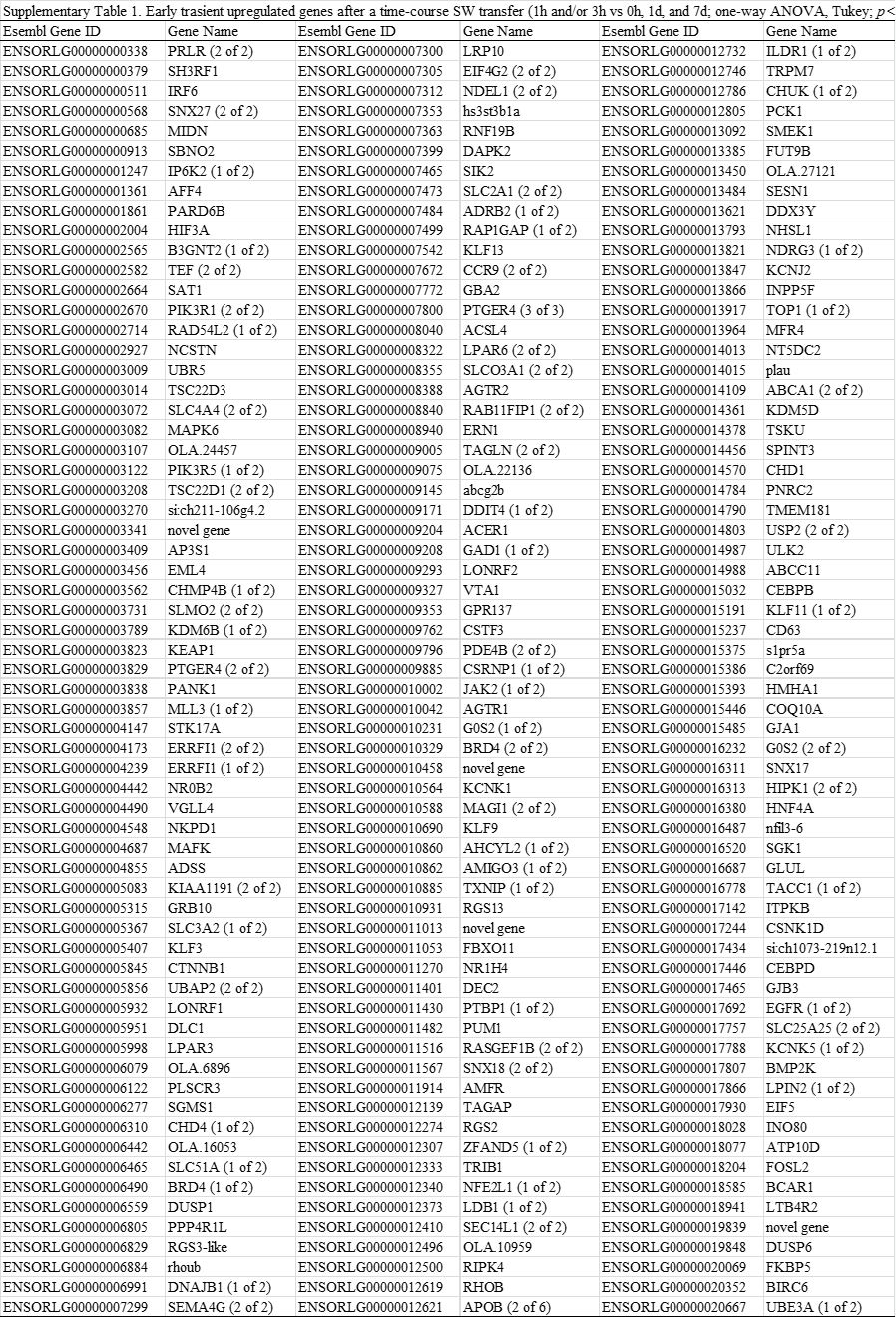

Supplement: Supplementary file 1 — Additional file 1: Table S1: Early transient upregulated genes after a time-course SW transfer (1 h and/or 3 h vs 0 h, 1d, and 7d; one-way ANOVA, Tukey; p < 0.05). (TIFF 646 KB) [file 12864_2014_6879_MOESM1_ESM.tiff]

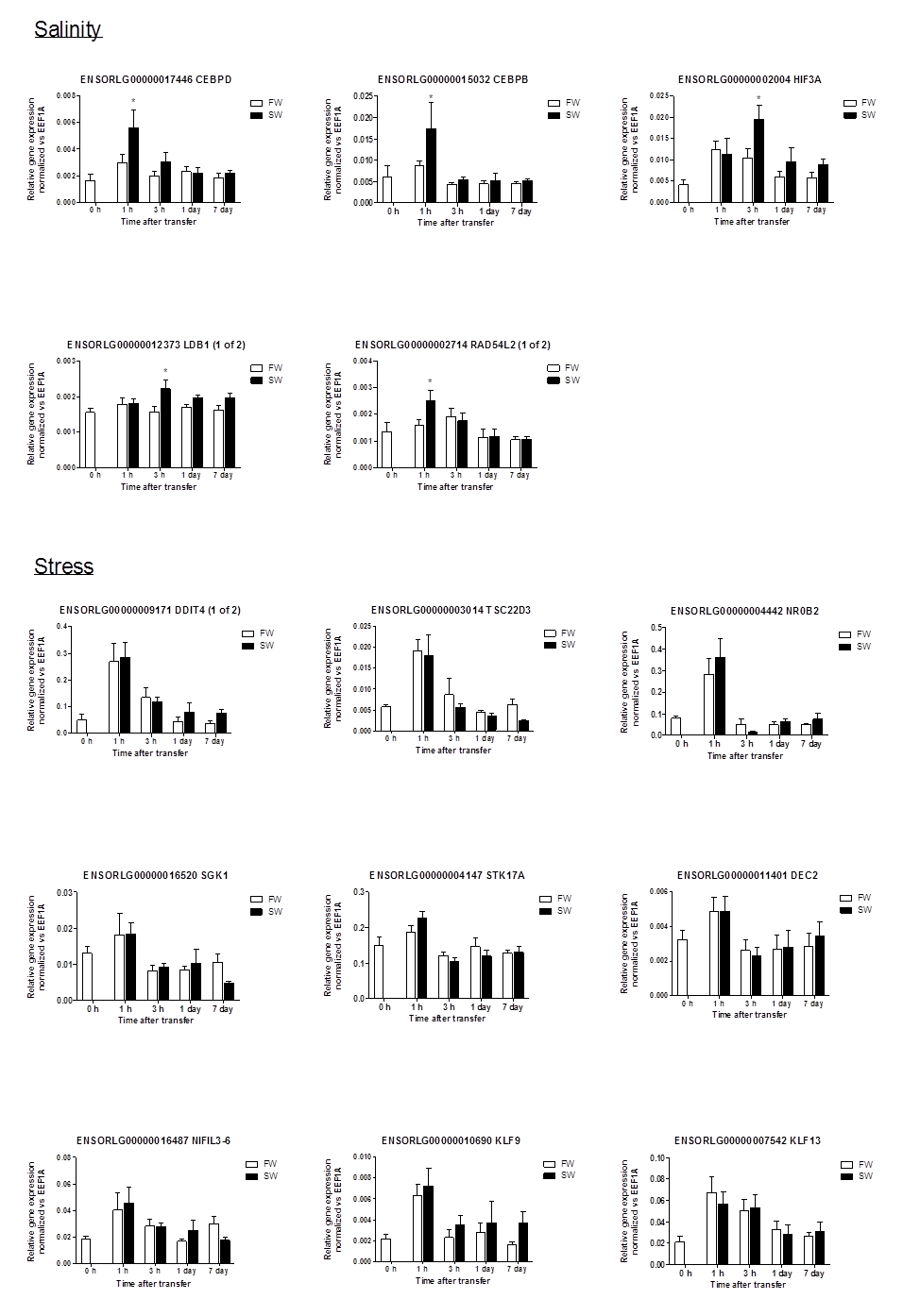

Supplement: Supplementary file 2 — Additional file 2: Figures S1-S4: Real time PCR results of the transcription factors in medaka intestine discovered from transcriptome. The expression patterns were categorized to salinity-related, stress-related, and insignificant changes. Statistical significant groups are indicated by asterisks in the bar graphs (two-way ANOVA, Bonferroni with time-matched comparison.*p < 0.05). (ZIP 666 KB) [file 12864_2014_6879_MOESM2_ESM.zip › 5418616931286175_MOESM1_ESM.tiff/figure S1.tiff]

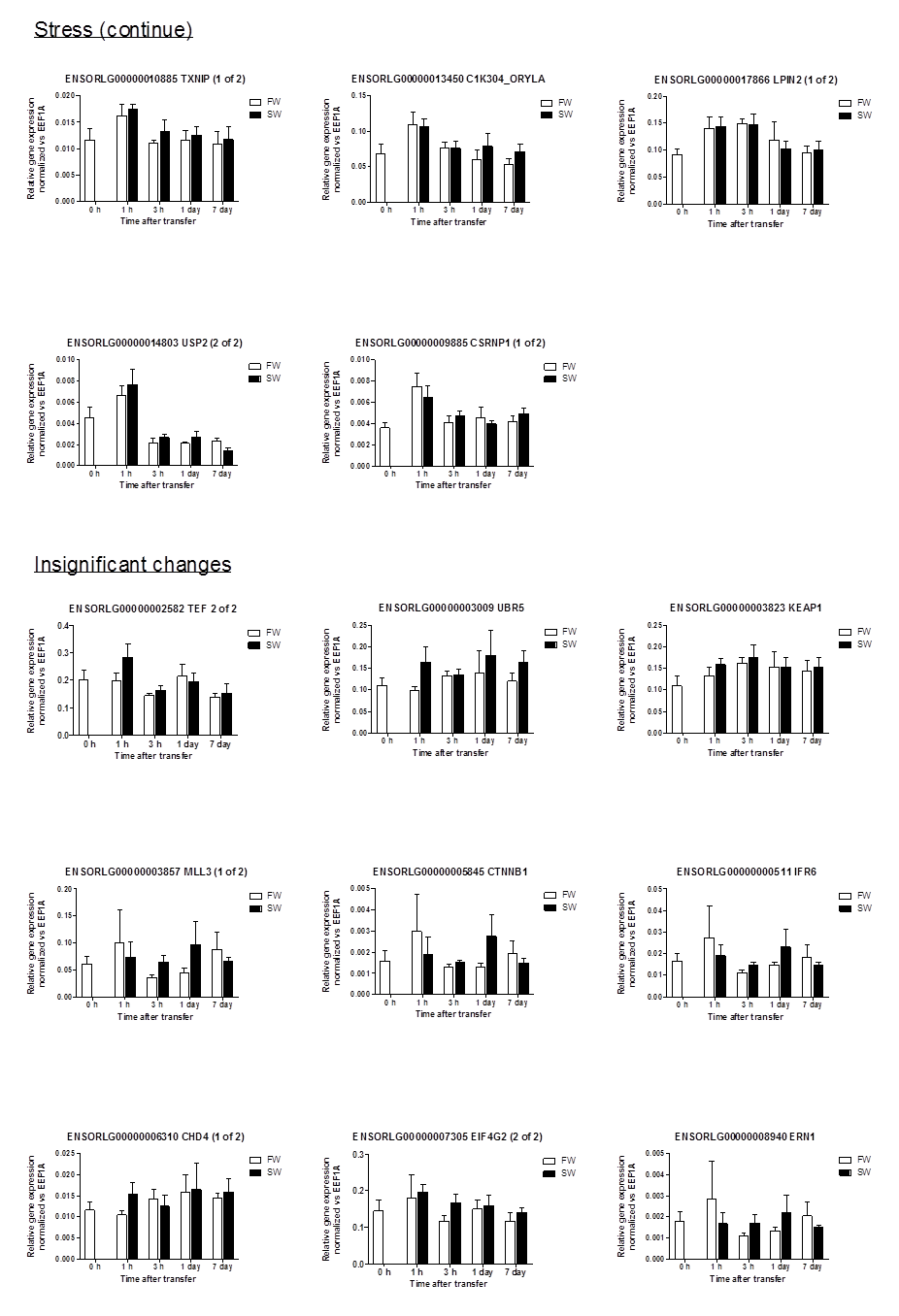

Supplement: Supplementary file 2 — Additional file 2: Figures S1-S4: Real time PCR results of the transcription factors in medaka intestine discovered from transcriptome. The expression patterns were categorized to salinity-related, stress-related, and insignificant changes. Statistical significant groups are indicated by asterisks in the bar graphs (two-way ANOVA, Bonferroni with time-matched comparison.*p < 0.05). (ZIP 666 KB) [file 12864_2014_6879_MOESM2_ESM.zip › 5418616931286175_MOESM1_ESM.tiff/figure S2.tiff]

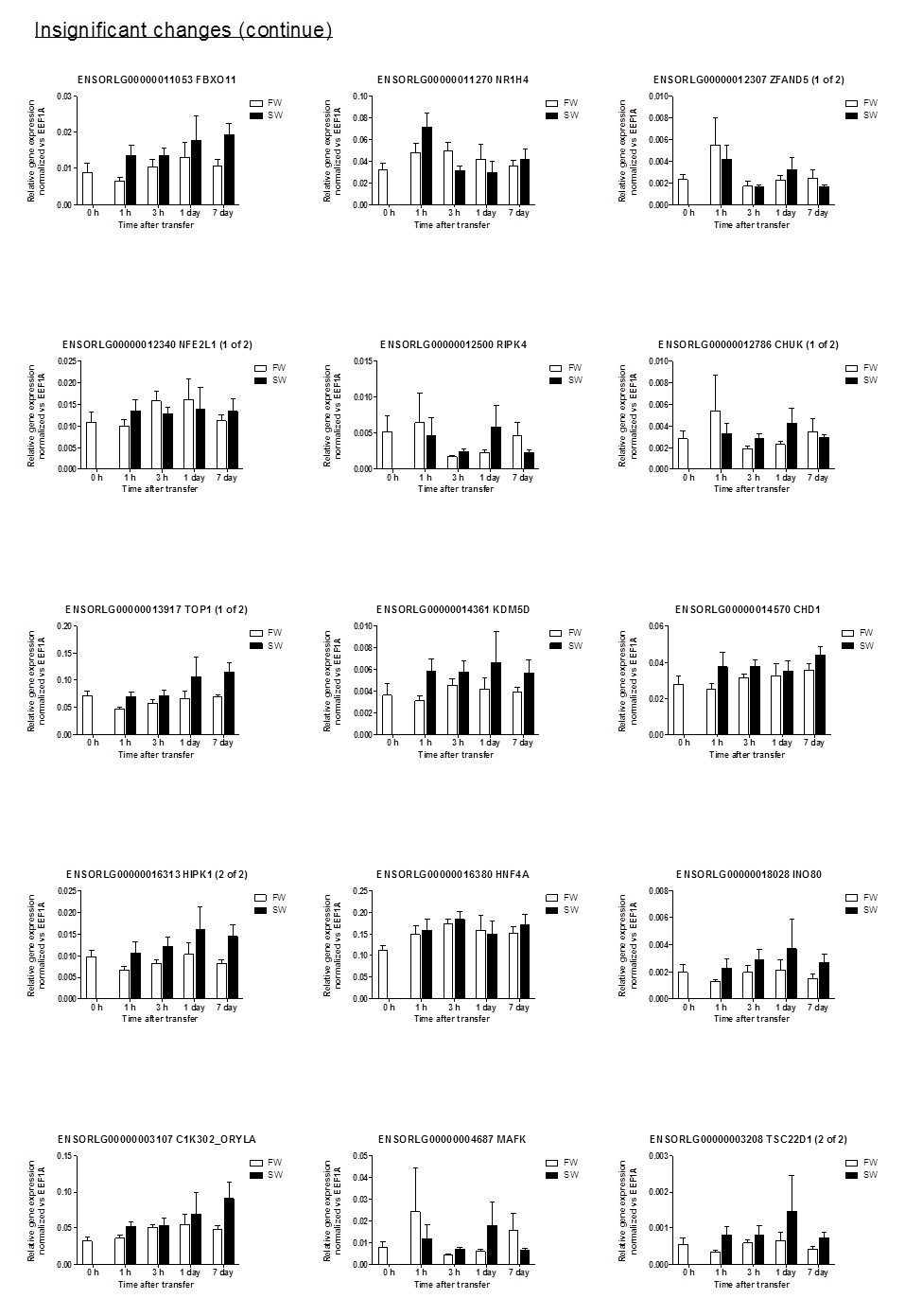

Supplement: Supplementary file 2 — Additional file 2: Figures S1-S4: Real time PCR results of the transcription factors in medaka intestine discovered from transcriptome. The expression patterns were categorized to salinity-related, stress-related, and insignificant changes. Statistical significant groups are indicated by asterisks in the bar graphs (two-way ANOVA, Bonferroni with time-matched comparison.*p < 0.05). (ZIP 666 KB) [file 12864_2014_6879_MOESM2_ESM.zip › 5418616931286175_MOESM1_ESM.tiff/figure S3.tiff]

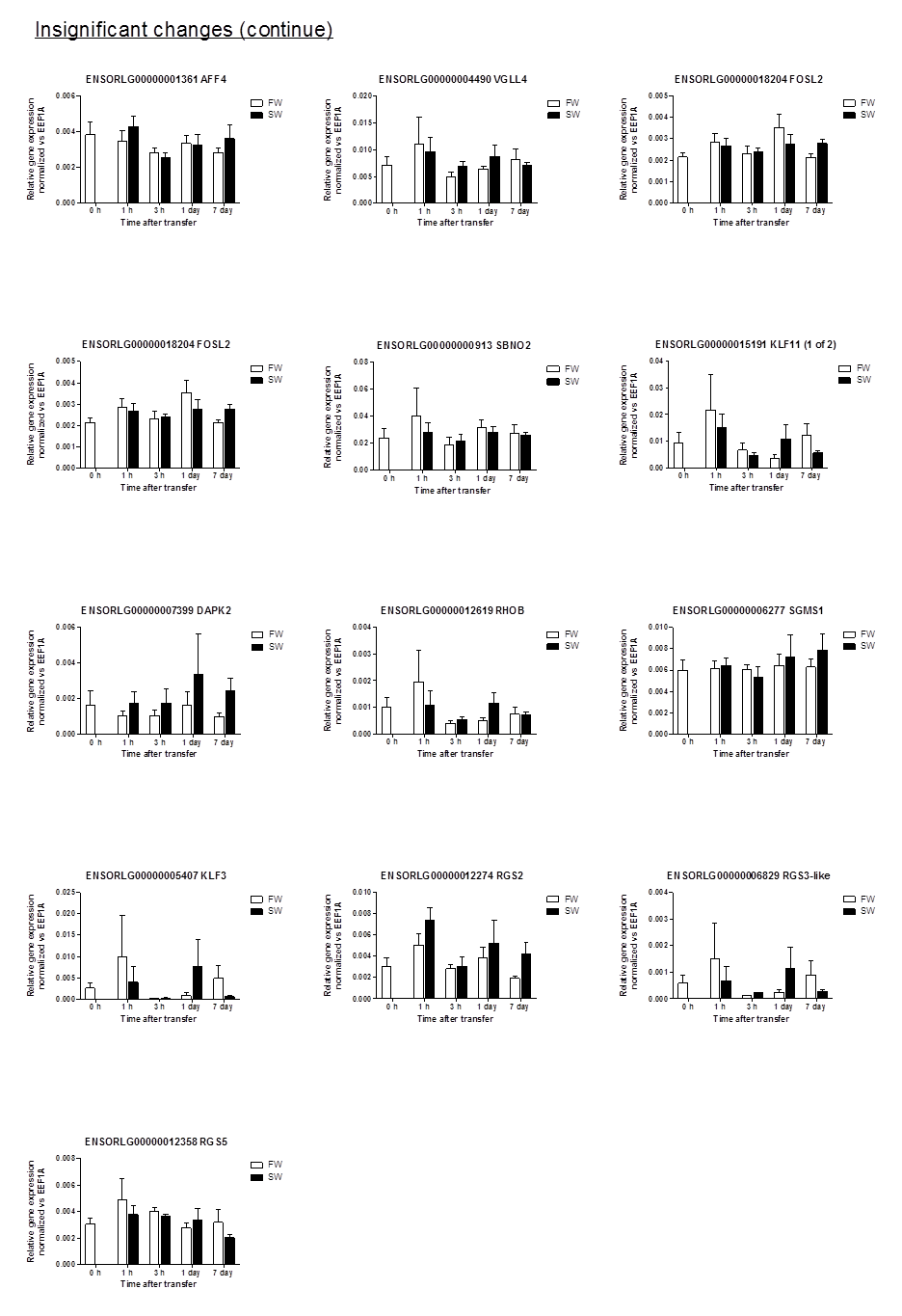

Supplement: Supplementary file 2 — Additional file 2: Figures S1-S4: Real time PCR results of the transcription factors in medaka intestine discovered from transcriptome. The expression patterns were categorized to salinity-related, stress-related, and insignificant changes. Statistical significant groups are indicated by asterisks in the bar graphs (two-way ANOVA, Bonferroni with time-matched comparison.*p < 0.05). (ZIP 666 KB) [file 12864_2014_6879_MOESM2_ESM.zip › 5418616931286175_MOESM1_ESM.tiff/figure S4.tiff]

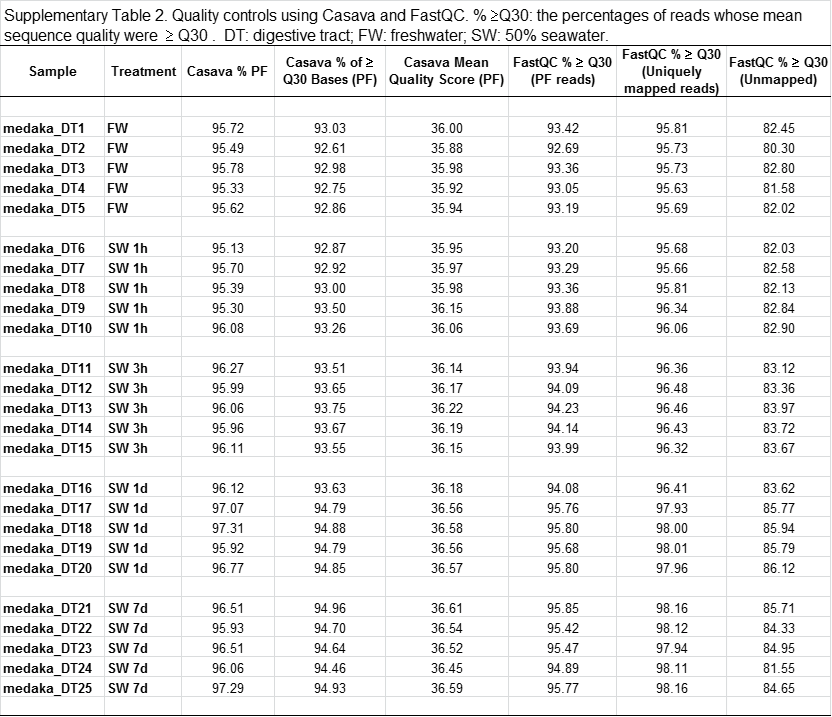

Supplement: Supplementary file 3 — Additional file 3: Table S2: Quality controls using Casava and FastQC. % ≥ Q30: the percentages of reads whose mean sequence quality were ≥ Q30. DT: digestive tract; FW: freshwater; SW: 50% seawater. (TIFF 209 KB) [file 12864_2014_6879_MOESM3_ESM.tiff]

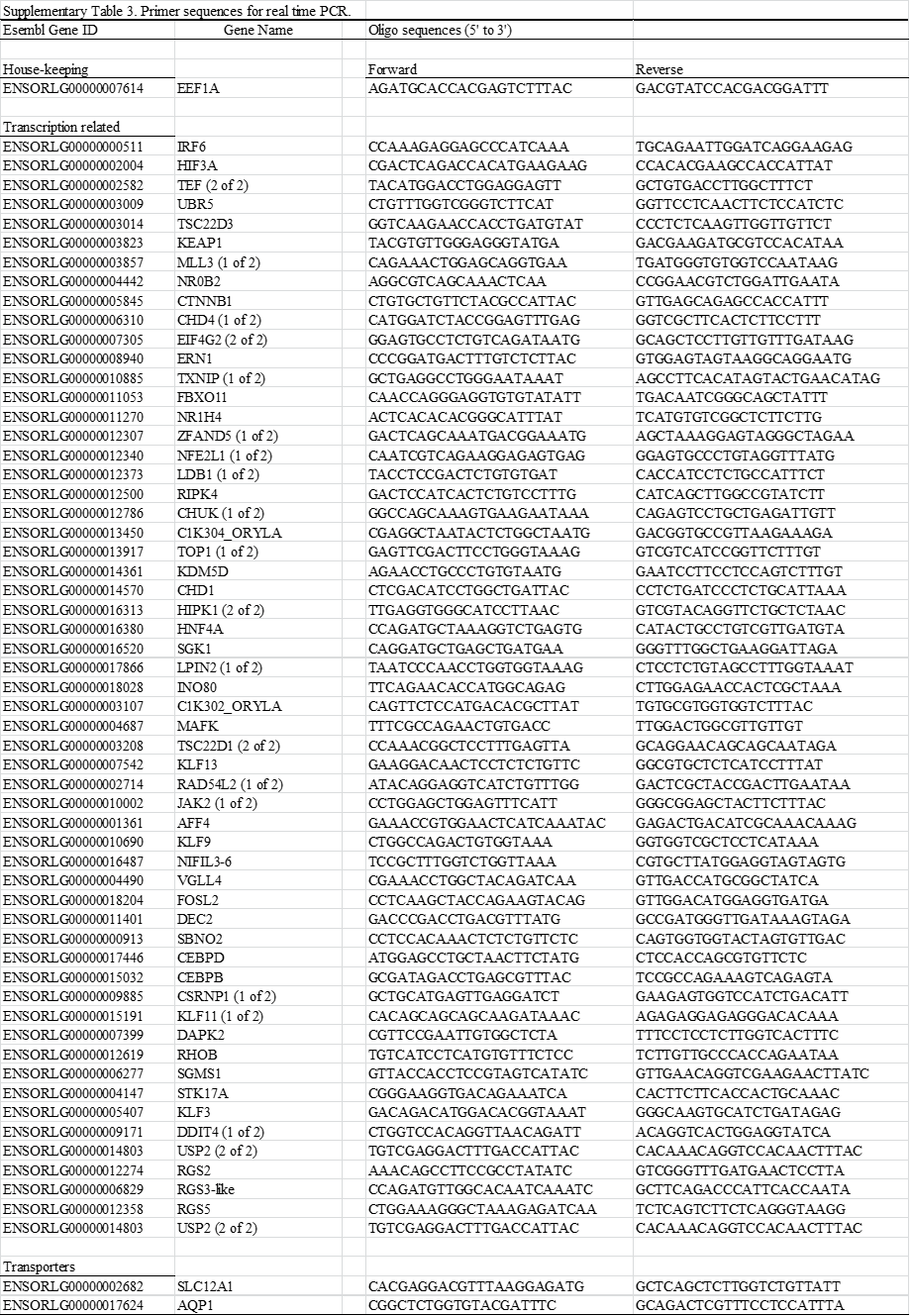

Supplement: Supplementary file 4 — Additional file 4: Table S3: Primer sequences for real time PCR. (TIFF 546 KB) [file 12864_2014_6879_MOESM4_ESM.tiff]
